# Rare Fetus-in-Fetu: Experience From a Large Tertiary Pediatric Referral Center

**DOI:** 10.3389/fped.2021.678479

**Published:** 2021-05-24

**Authors:** Mao Xiaowen, Cheng Lingxi, Lin Song, Pan Shengbao, Yang Xiaohong, Yang Xinghai

**Affiliations:** ^1^Department of Pediatric Surgery, Maternal and Child Health Hospital of Hubei, Tongji Medical College, Huazhong University of Science and Technology, Wuhan, China; ^2^Department of Radiology, Maternal and Child Health Hospital of Hubei Province, Tongji Medical College, Huazhong University of Science and Technology, Wuhan, China; ^3^Department of Ultrasonography, Maternal and Child Health Hospital of Hubei Province, Tongji Medical College, Huazhong University of Science and Technology, Wuhan, China

**Keywords:** fetus-in-fetu, pediatric, case series, rare disease, treatment

## Abstract

**Objective:** Fetus-in-fetu (FIF) is an extremely rare disease, and most prior publications are single case reports. Here, we describe the clinical characteristics, imaging manifestations, and the treatment and related complications of FIF from a large tertiary pediatric referral center.

**Materials:** After institutional review board approval, patients with a diagnosis of FIF between January 2010 and November 2019 were further selected and reexamined. We analyzed the general clinical characteristics, imaging manifestations, treatment, and prognosis of the patients.

**Results:** A total of seven (four male and three female) patients with FIF were included in the study. All patients were diagnosed with FIF during the antenatal ultrasound examination along with an abnormal increase in alpha fetoprotein, and it was confirmed by subsequent pathological examination. The median gestation period when FIF was first diagnosed was 25 (range: 22–32) weeks. Ultrasound, computed tomography, and magnetic resonance imaging were the main pre-operative diagnostic techniques used. All patients underwent FIF resection within 1 month after birth: four patients had open surgery and three had laparoscopic surgeries (one case was converted to open surgery); only one patient developed ascites after surgery. All patients are growing up healthy and without tumor recurrence at the last follow-up. The level of alpha fetoprotein decreased to normal within 1 year (range 3-10 months) after surgery performed.

**Conclusion:** As the size of the FIF increases, it can be found and diagnosed in antenatal ultrasound examination. Surgery is an important curative treatment for FIF and generally results in excellent long-term quality of life.

## Introduction

Clinically, fetus-in-fetu (FIF) is a very rare disease (1) and mostly occurs in neonates, with an incidence of about 1 in 500,000 live births; it is rare in adults (2). The pathogenesis of FIF is still controversial (3). At present, the two most common theories are the teratoma theory and the identical twin theory. According to the teratoma theory, FIF is a specialized form of teratoma with well-differentiated, highly organized, and mature organs. It shows organ differentiation, including the original heart, brain, eyeballs, intestines, and limbs, and thus, FIF is considered a highly differentiated teratoma (4). It has also been reported that teratomas and FIF appear at the same time. De Lagausie et al. (5) suggested that FIF and teratomas are not two absolute entities, rather the same pathological phenomenon at different stages of differentiation and maturity, with a likely overlap between the two. In part, their relationship remains to be further explored. Another hypothesis is that FIF is due to changes in the twinning process. Normal twins are deformed as symmetrical conjoined twins. FIF might result from abnormal embryogenesis in a diamniotic monochorionic twin pregnancy (4, 6). In the early stage of embryonic development (blastocyst stage) of the fertilized egg, the totipotent cell cluster in the blastocyst divides into two or more cell clusters. If these cell clusters develop normally, they will result in normal twins or multiple births. However, if the two embryos in a twin pregnancy are of different sizes, the larger one gets sufficient placental blood supply and continues to develop and become a normal fetus, while the smaller one remains underdeveloped owing to unfavorable *in utero* conditions and is encased in the normal twin during development.

There is usually one twin FIF, but a range of 2–5 FIFs has also been reported (7, 8). Although the FIF has no independent living ability, it is still a living tissue in the host. As its nutrient supply is derived from the host, it continues to grow with the host. FIF often show characteristic human development with presence of organs, tissues, and/or hair. They are mostly found in the retroperitoneum, but can also be found in the abdominal cavity, cranial cavity, sacrum, mediastinum, scrotum, oral cavity, kidneys, and even as cryptorchidism (1, 9–11). Clinically, it typically manifests as abdominal distension and a painless mass in the abdomen. If the mass is large, it produces compression symptoms. The diagnosis needs to be differentiated from that of a teratoma. Surgical resection is the main treatment for FIF and can likely offer a cure (2).

With advancements in imaging technology and the improvement of physicians' clinical experience, many patients can get a preliminary diagnosis during antenatal examinations (11, 12), and further diagnosis can be made by pathological examination of surgical specimens. Because the current reports on FIF mainly focus on single case reports (12–15), we retrospectively analyzed the clinical characteristics, imaging manifestations, treatment methods, and related complications and prognosis of FIF diagnosed in a single tertiary hospital since 2010 to improve the understanding of diagnosis and treatment of FIF.

## Patients and Method

This study was approved by the Maternal and Child Health Hospital of Hubei Province, Tongji Medical College, Huazhong University of Science and Technology Ethics Committee (Wuhan, China). Written informed consent was obtained from all patients after a full explanation of the procedure, and patient records and/or information were de-identified and anonymized before analysis.

Our study included all patients diagnosed with FIF from January 2010 to November 2019. All patients underwent some or all pre-operative imaging examinations [radiography, or Doppler ultrasound, or computed tomography (CT), or magnetic resonance imaging (MRI)]. After the initial diagnosis, all patients were treated by an experienced pediatric surgeon (YX), and the diagnosis was later confirmed by pathological examination. Patients pathologically diagnosed with teratoma and those who were not diagnosed by pathological examination during induction of labor were excluded.

Doppler ultrasonography (or other imaging above) was performed 1, 3, 6, 12, and 24 months after surgery to monitor the recurrence of disease and treatment effect, as well as the occurrence of surgical complications. In addition, alpha-fetoprotein (AFP) was performed 1, 3, 6, and 12 months after surgery considering the possibility of malignant present of the disease; 1 year after surgery, patients were advised to follow up once per year. Follow-up data in our study were mainly based on the review of patients' post-operative visit records at our hospital and the patient's examination report at other hospitals. The patient can communicate with the team of surgeon (YX) through WeChat, mobile phone, etc.

All data are stored in the medical records system of our hospital and can be recalled and viewed at any time after obtaining access from the hospital authority. Two clinicians (MX and CL) independently completed data collection and screening and compared them. For items with differences, the surgeon will make a ruling to ensure the accuracy of the data. The main purpose of our study was to descriptively analyze the clinical and imaging characteristics of FIF and report the effects of surgical treatment, complications, and prognosis.

## Results

**Figure 1:**
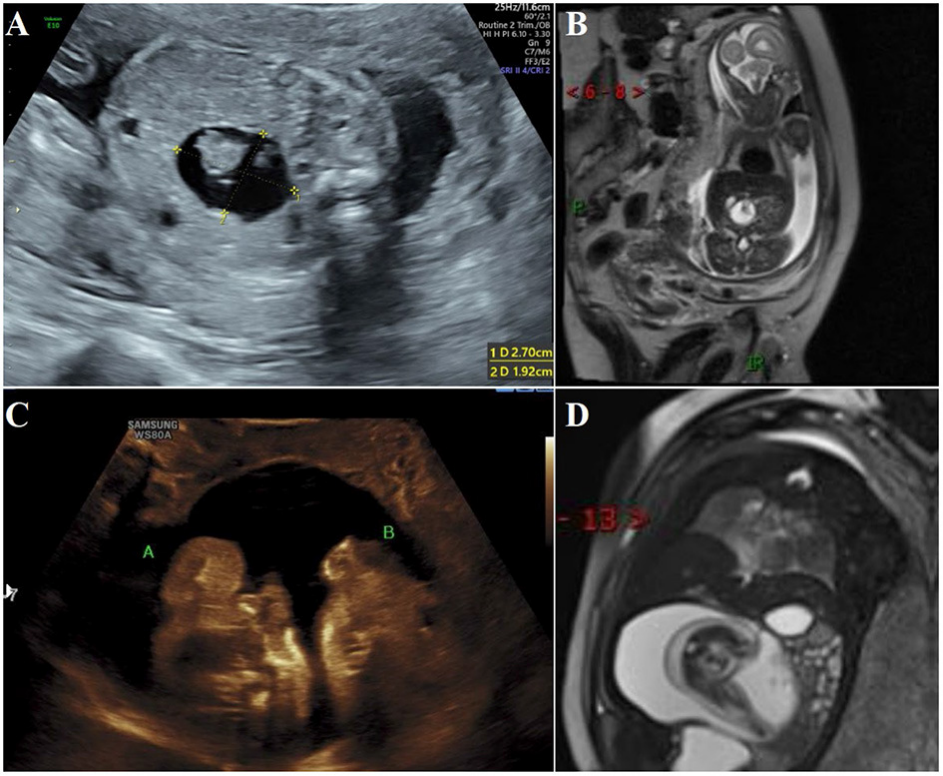
During antenatal examination, the parasitic fetus was found by ultrasound and MRI. **(A)** Ultrasound examination at 22 gestation weeks showed mixed density shadow of the right upper abdomen of the fetus; **(B)** MRI at 22 gestation weeks showed a long T1 long T2 signal foci measuring 2.4 × 1.9 cm near the midline area of the right upper abdomen of the fetus. The boundary was clear and the shape was regular. There are short cord-like short T2 signal foci in the lesion; **(C)** At the ultrasound examination at 37 gestation week, two parasitic fetuses were found; **(D)** MRI at 37 gestation weeks showed a round long T1 long T2 signal focus measuring 11.1 × 8.7 × 6.4 cm near the midline area of the right upper abdomen of the fetus, with clear boundaries and regular morphology. Fetal structures can be seen in the lesion, and adjacent tissues are compressed.

**Figure 2:**
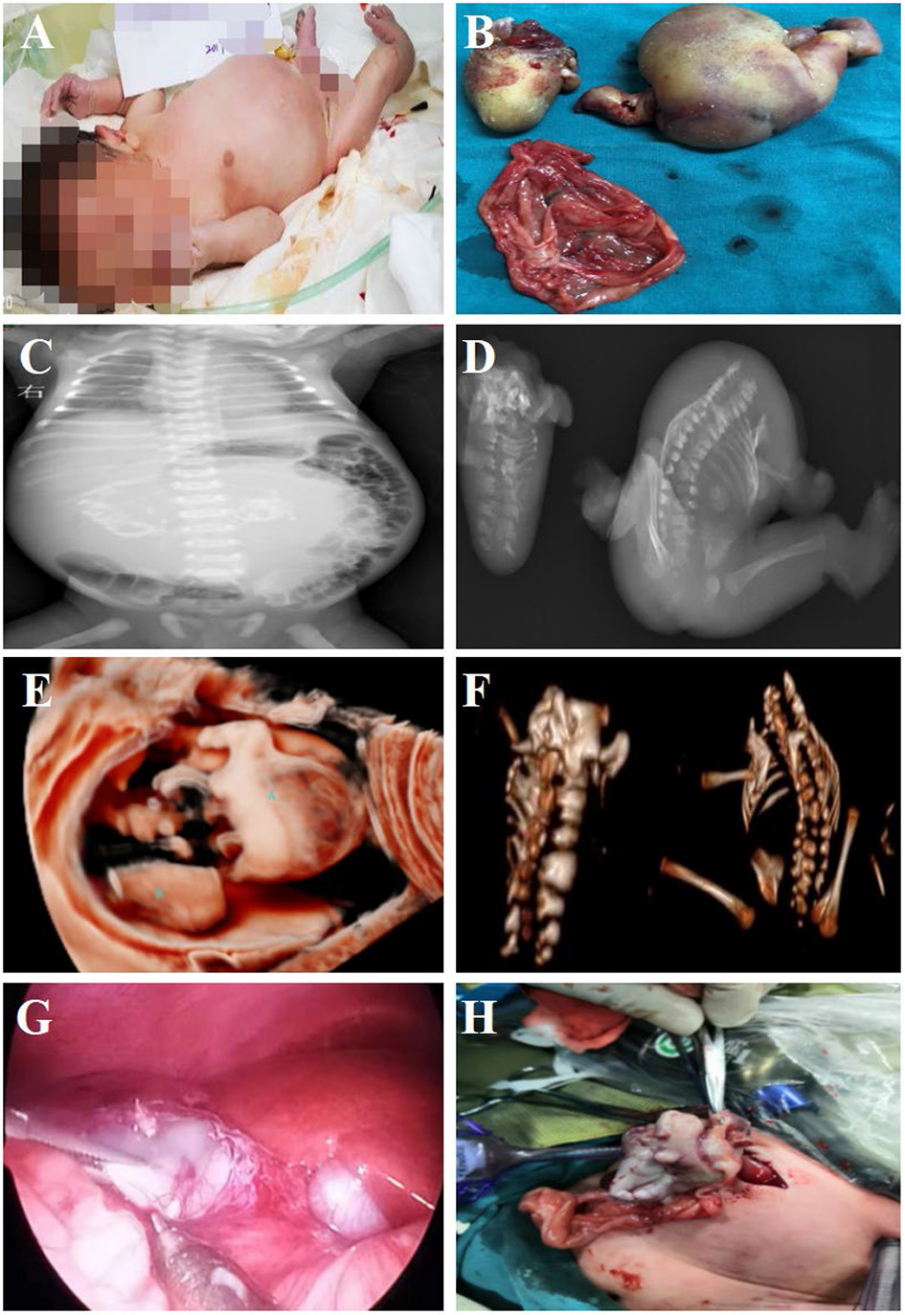
Fetus-in-fetu. **(A)** Patients with abdominal FIF present with abdominal bulging; **(B)** Specimen manifestations after FIF resection of the abdominal cavity; **(C)** Radiographic examination showed a bone-like shadow in the abdominal cavity; **(D)** Radiographic examination of FIF after operation; **(E)** Three-dimensional ultrasonic testing for FIF; **(F)** CT three-dimensional reconstruction technology to detect FIF after excision of the FIF; **(G,H)** The characteristics of FIF under laparoscopy, and it was extracted by enlarging umbilical incision.

Using the medical record system to screen all patients in our hospital in the past 10 years, a total of seven infants (4 male and 3 female) diagnosed with FIF were included in our analysis (Table 1). Family history was negative for congenital malformations, and there was no history of medication and drug use during pregnancy. Fetal abdominal masses were found during the antenatal examination of the patients' mother; the imaging findings are shown in Figure 1. The median gestation period when the fetal mass was first detected was 25 (range: 22–32) weeks. As the pregnancy progressed, the masses also increased; hence, FIF may not be detected early in pregnancy. Post-birth, abdominal bulging and distention were found upon physical examination in patients with large tumors that compressed the surrounding tissues (Figure 2A). Of all seven patients, two patients showed two FIFs in the abdomen (post-operative gross specimens of double FIF are shown in Figure 2B, while the remaining five patients only had one FIF. The FIF of all patients were located retroperitoneally.

**Table 1:** Summary of characteristics of all seven fetus-in-fetu cases.

**Case**	**Sex of** **patient**	**Diagnosis time**	**No. of** **fetus-in** **-fetu**	**Age at** **operation** **after birth**	**Laboratory** **examination** **(pre-operative)**	**Site**	**Surgery** **type**	**Ultrasound findings**	**Radiographic findings**	**CT or/** **and MRI** **findings**	**Gross anatomy**	**Follow-up**
One	Male	Antenatal/24 weeks of pregnancy	1	4 days	AFP > 3,000 ng/mL	Retroperitoneal	Open surgery	Cystic components with mixed density mass, with bone imaging manifestations and umbilical cord blood flow manifestations	Skull and spine-like high-density shadows are vaguely visible on the left side of the mid-abdomen	CT: A huge mixed-density space-occupying lesion (5.8 × 5.9 × 7.5 cm) can be seen on the left side of the upper mid-abdomen, with clear boundaries, and the skull, spine, and skeletal shadows of the limbs, accompanied by shadows of soft tissue, fluid and fat density, are next to the stomach, pancreas, spleen. The enhanced scan showed local enhancement, and the branch vessels of the superior mesenteric artery entered the lesion.	Umbilical cord, amniotic membrane, spine, lower limbs, feet, intestine	1. No diseases recurrence and occurrence of complications. 2. AFP normal information was missed.
Two	Male	Antenatal/22 weeks of pregnancy	2	10 days	AFP > 5,400 ng/mL CEA: 1.4 ng/mL CA125: 6.2 U/mL	Retroperitoneal	Open surgery	Cystic components with mixed density mass with bone imaging manifestations and umbilical cord blood flow manifestations	Massive soft tissue shadows can be seen in the middle of the abdomen, bone-like shadows can be seen inside, and the bowel is pushed	MRI: oval-shaped long T1 long T2 signal foci, with clear boundaries measuring 12.4 × 7.6 × 8.9 cm in size, and fetal-like structures can be seen inside, and there is no obvious enhancement.	One: amniotic membrane, spine, up and lower limbs, feet, external genitalia Another: amniotic membrane, spine	1. No diseases recurrence. 2. Occurrence of complication of ascites one months after surgery. 3. AFP normal in 3 months after surgery performed
Three	Female	Antenatal/25 weeks of pregnancy	2	8 days	AFP: 5,400 ng/mL CEA: 1.52 ng/mL CA125: 11.8 U/mL	Retroperitoneal	Open surgery	Cystic components with mixed density mass. Bone imaging manifestations and blood flow signals in the mass	The abdomen is obviously swollen, the left side of the abdominal cavity is high-density, and the distribution of intestinal gas is uneven	CT: A huge cystic solid mass on the left side of the abdominal cavity (9.2 cm× 8 cm× 6.6 cm), which occupies the left abdominal cavity. The skull, spine, and limbs are visible in the mass, and the surrounding tissues are obviously compressed	One: Amniotic membrane, spine, lower limbs, feet, intestine Another: Amniotic membrane, spine, cartilage	1. No diseases recurrence and occurrence of complications. 2. AFP normal information was missed.
Four	Female	Antenatal/30 weeks of pregnancy	1	26 days	AFP:1905 ng/mL	Retroperitoneal	Laparoscopic surgery	Cystic components with mixed density mass	None performed	CT: A solid cystic mass (5.4 × 6.2 × 5.8 cm) in the upper right abdomen, with clear borders, bone-like structures visible inside, enhanced vascular-like enhancement, small and partially visible blood vessels	Amniotic membrane, spine, lower limbs, feet, intestine, external genitalia	1. No diseases recurrence and occurrence of complications. 2. AFP normal in 3 months after surgery performed.
Five	Male	Antenatal/32 weeks of pregnancy	1	24 days	AFP: 15,625 ng/mL CEA: 5.0 ng/mL CA125: 8.5 U/mL	Retroperitoneal	Open surgery	Cystic components with mixed density mass	None performed	CT: A solid cystic mass in the upper left abdomen (5.0 × 5.4 × 5.2 cm), with clear borders; irregular high-density and slightly low-density shadows can be seen inside. The surrounding kidneys and intestines are compressed and pushed forward. The supply vessel of the tumor is the abdominal aorta.	Amniotic membrane, lower limbs, liver, intestine	1. No diseases recurrence and occurrence of complications. 2. AFP normal information was missed.
Six	Female	Antenatal/26 weeks of pregnancy	1	10 days	AFP > 3,000 ng/mL CEA: 2.7 ng/mL CA125: 8 U/mL	Retroperitoneal	Laparoscopic surgery	Cystic components with mixed density mass. Spine imaging shadow	Multiple irregular calcifications in the upper right abdomen	MRI: Cystic-solid mixed-density space-occupying lesions (7.5 × 7.6 × 8.7 cm), some solid lesions are slightly enhanced on the enhanced scan, and the blood vessel of the tumor comes from the branch of the abdominal aorta	Amniotic membrane, head, hair, cartilage, spine, lower limbs, feet	1. No diseases recurrence and occurrence of complications. 2. AFP normal in 6 months after surgery performed.
Seven	Male	Antenatal/25 weeks of pregnancy	1	11 days	AFP > 3,000 ng/mL CA125: 1.2 ng/mL CA125: 7.1 U/mL	Retroperitoneal	Switched from laparoscopic surgery to open surgery	Cystic components with mixed density mass	Intestinal gas on the left side is unclear, mostly distributed on the right side	CT: High- and low-density masses (5.2 × 5.5 × 5.0 cm) can be seen in the upper abdomen, and long tubular bone structures and spine-like structures can be seen inside. The blood supply vessel of the tumor is the branch of the superior mesenteric artery	Amniotic membrane, renal, cartilage, intestine	1. No diseases recurrence and occurrence of complications. 2. AFP normal in 10 months after surgery performed.

*CT, computed tomography; FIF, fetus in fetu; MRI, magnetic resonance imaging; AFP: alpha fetoprotein; CEA: carcinoembryonic antigen; CA125: carbohydrate antigen 125*.

All neonates were reexamined by ultrasound after birth, and all showed cystic masses with mixed density shadows. The tumors had clear boundaries with surrounding tissues and organs, accompanied by bone and spine imaging shadows (Figures 1A,C). Radiographs mainly showed the shadow of the soft tissues of the abdomen, wherein the intestinal tract was pushed, and the distribution of intestinal gas appeared uneven, along with bone-like shadow (Figure 2C). Radiographic examination of the FIF removed after surgery was performed to further confirm the diagnosis (Figure 2D). It is difficult to diagnose the FIF radiographically in patients without axial bones, but it can be well-obtained for bone structures. The cross-section of the CT showed an elliptical mixed density mass with a clear boundary, and the bone, soft tissue density, and fat density, and other structures were seen inside, the adjacent organ tube was compressed and displaced along with the gas in the intestines. Three-dimensional ultrasound technology is very intuitive to display FIF (Figure 2E). CT three-dimensional reconstruction technology can show the parasitic growth more clearly, along with a complete picture of the bony structure in the fetus. It can also show the anatomical relationship between the FIF and the surrounding bony structures (Figure 2F).

All patients underwent tumor resection surgery after birth, and the average age of patients at the time of surgery was 13 days (median: 10 days, range: 4–26 days). Two patients underwent complete laparoscopic surgery. One case was converted to open surgery to remove the FIF owing to heavy adhesion between the mass and surrounding tissues. The remaining four patients underwent open surgery from the beginning. Cystic space-occupying lesions were seen during the operation (Figures 2G,H). After the cystic fluid was slowly sucked out, the mass was bluntly separated, and the blood vessels supplying the tumor were carefully cut off. The post-operative pathological examination results were consistent with the pre-operative imaging diagnosis, and the diagnosis of FIF was confirmed. No serious complications occurred in any patient. One patient had ascites, which might be related to the early removal of the drainage tube after the operation. After puncture and drainage, all patients are alive and healthy without further signs of disease or recurrence. Further, all seven infants underwent pre-operative laboratory examination and had elevated AFP levels, but as the post-operative follow-up time was prolonged, AFP levels gradually returned to normal values with range 3-10 months after surgery (Table 1).

## Discussion

In the 19th Century, Meecker first described FIF as a rare phenomenon in which a fetus of deformed twins was parasitized in the abdominal cavity of its healthy partner (1). FIF is relatively rare in clinical practice, with most reports thus far being single case reports (9, 12–14, 16). Most FIF are obstructed in their organ development and thus manifest with anencephaly and/or asthenia. In general, the development of lower limbs is usually better than that of the upper limbs. The FIF can be parasitic on any part of the host's body, and the symptoms and signs caused by different parts are different. For example, obvious abdominal masses can cause pressure on the surrounding organs. Wu et al. reported cardiac arrest in a baby due to a huge FIF in the posterior mediastinum (16). Physical examination revealed that the abdomen was swollen, soft, and without tenderness, and masses were palpable in the abdomen. The texture was medium, the boundary was clear, the surface was smooth, and the mobility was poor. FIF and the host are often the same sex, the same blood type, and the chromosomal karyotype is usually normal and similar to the host. By gross and microscopic examination, the diagnosis of paragenesis is not difficult. The mass looks like a fetus, with limb-like structures, abnormal skeletal development, and intestinal development, as well as gonads and adrenal glands, notochord, kidney tissue and bladder, and other tissue structures, besides, the FIF is often enclosed in a thin fibrous capsule, covered with a single layer of epithelium or squamous epithelium (17).

Imaging examinations play a key role in the diagnosis of FIF (11, 15). With the development and popularization of ultrasound, radiography, CT, and MRI, most FIF can be diagnosed during antenatal and before surgery, and all the cases we reported were fully diagnosed during antenatal examinations. After the operation, the pressure of the tumor on the surrounding organs was alleviated and related symptoms and complications were relieved. In particular, the examination found that the axial bones (cranium, spine, sternum, ribs) in the lesions are key to the diagnosis of FIF. Not all FIF has a distinguishable spine imaging, and patients can also be diagnosed as a FIF even if there is no spinal structure but the limbs are well-developed. When the parasitic fetal tumor is large, the surrounding organs are compressed and displaced, it can be seen during X-radiography as space-occupying lesions casting a shadow of soft tissue density and mass. However, the density resolution of plain X-ray film is low, and the FIF is buckled in the body. The images of retroperitoneal organs, intestines, fat, etc. are overlapped, and the parasitic fetal bone is not fully calcified; hence, it is difficult to distinguish the spinal images at this time. In our case, some radiographic examinations did not show any skeletal shadows. However, CT examination could detect that the FIF presented as mixed density shadows, irregular shapes, and clear boundaries. The density shadows of fluid, fat, bone, and soft tissue were seen in the FIF. In addition, CT three-dimensional reconstruction can completely display the axial bone system in the FIF, facilitating direct diagnosis and providing a basis for clinicians for an intuitive, true, and reliable imaging diagnosis. Both radiographic and CT examinations have the disadvantage of radiation exposure. Ultrasound examination is not only a very safe prenatal examination but also can be used for the diagnosis of prenatal and neonatal FIF. It is worth noting that although ultrasound examinations are safe, convenient, and economical, FIF and teratoma have similar sonographic features on ultrasound examination, and hence, the risk of misdiagnosis is present. In recent years, MRI has also been used to diagnose FIF, which can clearly identify the soft tissues and organs surrounding the FIF, thereby providing valuable imaging data for the formulation of surgical strategies (15). Compared with CT, MRI is an ideal imaging modality with inherent high tissue contrast and spatial resolution. In addition, MRI avoids the need for iodine contrast and eliminates the risk of ionizing radiation, it is considered safe in pregnancy (15).

There are certain difficulties in the differential diagnosis of FIF and teratoma, both showed space-occupying lesions and masses (18). In terms of pathomorphology, FIF is composed of well-developed fetal organs, spine, or limbs. Teratoma is a dysplasia and solid tumor composed of three germ layers: outer, middle, and endoderm. It can be differentiated and mature. Cartilage or teeth, single or multiple cysts can be seen on the cut surface, and the cysts may contain sebum or mucus. The majority of FIF are located in the retroperitoneal space of the abdomen; FIFs in other parts are rare (9). FIF are covered with skin and hair, have a clear source of blood supply similar to the structure of the umbilical cord, and are located in the amniotic sac, which can stop growing due to insufficient blood supply. The most common sites for teratoma are ovaries, testes, posterior peritoneum, and the sacrococcygeal region. In imaging studies. FIFs appear as formed vertebrae or limb bones, while teratomas were scattered irregular calcifications or ossifications, without formed vertebrae and limb bone structures. When the diagnosis is difficult, the application of imaging (such as CT-based three-dimensional reconstruction) can help the diagnosis and differential diagnosis of FIF and teratoma. Moreover, the rare mature teratoma-fetal teratoma, which is occasionally located in the ovary, can also be found in the skull, orbits, ribs, and pubic bones.

Once the FIF is diagnosed, surgical treatment is essential, and the prognosis is good after complete resection (2). The success of the resection is related to the size of the mass, local pressure, intracystic mass bleeding, and other uncertain diagnoses. The FIF is often enclosed in a thin fibrous capsule, covered with a single layer of epithelium or squamous epithelium. This feature of the FIF makes the tumor and surrounding tissues and organs clear, and it is easy to bluntly peel off the tumor without damaging the surrounding organs and increasing complications. The embryo is removed together with the cyst to avoid leakage of the cyst contents. The FIF along with cyst could be completely removed during surgery in all seven patients in our series.

Surgical treatment of abdominal FIF can be considered through laparoscopy. Laparoscopy has the advantages of less trauma, clear vision, and beautiful appearance. At the same time, attention should be paid to avoid the tube during separation so that the adjacent organs remain undamaged during separation; further, the capsule and mass should be kept intact during separation and removal.

After surgery, the patient should be carefully monitored. Follow-up evaluation of AFP or β-human chorionic gonadotropin (β-HCG) levels combined with imaging can be used for monitoring and close clinical observation. Although the various components of the FIF are mature, there is still the possibility of malignant transformation. Hopkins et al. (19) reported that malignant transformation occurred after FIF resection. The pathological diagnosis was recurrence of an endodermal sinus tumor after FIF. Although the AFP and β-hCG levels in FIF may be elevated, persistent high levels are more likely to be a sign of malignant tumors. Tumor markers of AFP and β-HCG can be used as the basis for patient diagnosis, follow-up observation, and determination of malignant recurrence of FIF (13). All seven cases showed no diseases recurrence and the level of AFP decreased to normal within 1 year after surgery performed.

In conclusion, although FIF is a congenital malformation that is rarely encountered in clinical practice, it is potentially harmful to the human body, especially when the patient is a neonate. Early diagnosis is crucial, followed by complete surgical excision and long-term follow-up. The development of antenatal diagnostic techniques and the popularization of imaging technology have increased the diagnosis rate of FIF. Our study could help for in-depth understanding of the disease, and will improve the diagnostic accuracy and treatment, thereby increasing the cure rate of patients. The incidence of FIF has increased in recent years in our center, and it is necessary to conduct multi-institute research on the risk factors of FIF in the future to adopt targeted prevention, which might be a significant topic.

## Data Availability Statement

The original contributions presented in the study are included in the article/supplementary material, further inquiries can be directed to the corresponding author/s.

## Author Contributions

MX and YXin conceived the research and wrote the manuscript. MX, CL, LS, PS, and YXia collected the data. MX analyzed the data and prepare the figures and tables. All the authors were involved in the approval of the final version.

## Conflict of Interest

The authors declare that the research was conducted in the absence of any commercial or financial relationships that could be construed as a potential conflict of interest.
